# Terra Preta production from Ghanaian and Zambian soils using domestic wastes

**DOI:** 10.1038/s41598-024-75521-y

**Published:** 2024-10-15

**Authors:** Dora Neina, Bruno Glaser

**Affiliations:** 1 Department of Soil Science, School of Agriculture, College of Basic and Applied Sciences, University of Ghana, P.O. Box LG 245, Legon, Accra Ghana; 2 Department of Soil Biogeochemistry, Institute of Agricultural and Nutritional Sciences, Martin Luther University Halle-Wittenberg, von-Seckendorff-Platz 3, Halle (Saale), Germany

**Keywords:** Animal manure, Biochar, Charcoal residues, Kitchen wastes, Waste ash, Amazonian dark earths, Biogeochemistry, Environmental sciences

## Abstract

Quests for productive soils to close yield gaps call for innovative strategies. This study tested an off-site formation of the Amazonian Terra Preta (TP) in a potential modern analogon under coastal savannah climatic conditions of Ghana. Four Ghanaian and two Zambian soils; two types of biochar (i.e., rice husk biochar and charcoal residues); domestic wastes (i.e., kitchen leftovers, animal manures, human urine, and kitchen ash) were mixed with the soils wetted to 100% water holding capacity, and incubated under aerobic conditions for nine months. Indicators of the TP include total carbon (C), pH, base saturation, basic cations, and plant-available P, which were measured using standard methods of soil analysis. The TP formation enhanced soil pH by 0.02 to 2.9, ranging from pH 7.2 to 8.2, with charcoal residues having the highest effect on pH. The modern TP was characterized by relatively high total C, pH, K, Ca, Mg, Na, base saturation, and plant-available P. These properties reflect unique interactions between the chars, wastes, and soils, suggesting the potential for on-site TP formation. It calls for further studies, commitment, and perseverance in their formation in the future.

## Introduction

Soil health recognizes the potential functions of soils for sustainable ecosystems. A strive for sustainable management of soil systems to achieve resilience calls for holistic approaches such as traditional, non-traditional, scientific, and ancient techniques. Applying such approaches requires consciousness and persistence to obtain sufficient results for intensification^1,2^. One such strategy is the formation of Terra Preta (TP), sustainably fertile soils with stable soil organic matter (SOM) formed by the pre-Columbian population by enrichment with organic matter from the surroundings, food leftovers, excrements, and residues of smoldering fires such as charcoal and ash^3–5^. Due to its agricultural and environmental value, there were attempts in the past to replicate it under the name “Terra Preta Nova”. However, despite its attractive goals to enhance sustainability, forest conservation, and promote carbon (C) sequestration, the concept was overthrown by huge interests in biochar^2^, which is now a byword endued with an influential interdisciplinary touch. However, it should be clear that biochar is only one ingredient of TP and that large amounts of nutrients and soil microorganisms are also necessary for TP formation^4^.

Previous studies showed that TP comprised original soils, up to 14% SOM, with the total C containing up to 50% charcoal, potsherds, ash^6–8^, debris of bones, shells, and turtle carapaces^9^. The SOM comprises human excrements, animal manure, and plant debris^10,11^. It is proven that charcoal and the tremendous amounts of nutrients were the source of the high fertility of TP^2,10,12^ and, therefore, does not require any external fertilizer sources for good crop yields^13^. The fertility of TP represents sustainability in all aspects, as testified by farmers^7,14,15^ because it sustains food production and farmers’ livelihoods, reduces cropland expansion, enhances waste management, and contributes to biodiversity conservation. Crop yields on TP are about twice those of adjacent soils^10^.

Biochar (charcoal), a key ingredient of TP^3,4,10,16^, attracted the wide interest in biochar research. So far, almost 20,000 peer-reviewed research articles with biochar as title exist (about 1,000 reviews and about 100 quantitative reviews using the search term “biochar” as title in the ISI Web of knowledge scientific database. Therefore, there is unambiguous scientific evidence that biochar can improve most of the ecosystem functions such as biomass production^17^, C sequestration^18^, water storage capacity^19^, nutrient holding capacity^17^, nutrient availability^20^, greenhouse gas emissions^21^ and many others^22^. Generally, the application of pure biochar produces an additional mean yield increase of 25% in the tropics compared to virtually no extra crop yields in temperate regions^17,23^. This observation was remarkable in acid soils because of the liming effect of biochar, and the improved water holding capacity. The effect of biochar on soil properties seems to be more residual and additive, peaking after three years^24,25^ and thus requires repeated applications to achieve the best results. However, these effects are enhanced through plant nutrient use efficiency of biochar co-applications with inorganic or organic fertilizers^26^. This synergy produces nine-fold effects on crop yield and soil fertility for both organic and inorganic fertilizers^27^. Also, increasing the application rates of biochar (i.e., > 10 Mg ha^− 1^) can increase yields of over 200%^10^.

There is evidence that TP formation is driven by several factors: (a) It is a long-term process that takes thousands of years to develop compared to the direct application of biochar, which equally yields results similar to TP^2,6,15,28^. Indeed, time drives soil formation, as stated earlier in the proposed formation of TP^29^. (b) Terra Preta can form from different soil types^12,28^. (c) There is evidence that climatic conditions influence the formation of TP^28,30^, considering their current locations, e.g., Amazonia and humid West Africa, including Ghana^30,31^. (d) Further, an intense deposition of nutrient-rich materials is required for TP formation, as stated by the indigenes of Amazonia^15^. (e) The high fertility of TP is augmented by the unique properties of charcoal, which originated from traditional burning methods such as smoldering fires for spiritual or cooking purposes due to the lack of matches^10,12,32^. However, the properties of charcoal (today’s biochar) are known to be influenced by the type of feedstock and pyrolysis temperature^33–36^. Therefore, it was hypothesized that despite the time needed for TP formation, the other factors can be manipulated to have an overriding effect to form the TP Model soil proposed earlier^29^. Therefore, the objectives of this study were to (i) test the off-site formation of TP under coastal savannah agro-climatic conditions, and (ii) investigate the major properties of the original TP (Amazonian TP) based on characteristics of TP such as high total C, high cation exchange capacity (CEC) and high plant-available phosphorus (P) content. This study has relevance for soil health resilience, restoration, agricultural productivity, waste management and environmental sustainability.

## Results

### Properties of the original soils and organic soil amendments

#### Original soils

The properties of the original soils were characteristic of tropical soils, suggesting the need for improvement. Soil texture varied from fine with higher bulk densities associated with the dry season sampling for the urban garden soils to those with coarse texture and lower bulk densities (Table 1). The pH values ranged from acidic, slightly acidic, and neutral to slightly alkaline (Table 1). Soil organic carbon contents were generally < 2%, comprising four soils with < 1% and two soils had about 1.3 to 1.7% (Table 1). Urban garden soils had substantial amounts of plant-available P, mostly > 160 mg kg^− 1^, whereas the other soils had < 20 mg kg^− 1^ (Table 1). The ∆pH values grouped into three soils with negative values, while the other three soils had positive values (Table 2). The CEC of the soils formed a group involving the urban garden soils (Dzorwulu Vertisol and GBC Acrisol) with > 10 cmol_c_ kg^− 1^, Adansam Lixisol and Lukweta Acrisol with values > 7 cmol_c_ kg^− 1^, and Chitotokoloki Arenosol and Dompem Acrisol with ≥ 4 cmol_c_ kg^− 1^ (Table 2). The magnitude of the exchangeable cations was in the order Ca > Mg > Na > K in four of the soils and Ca > Mg > Na = K in two of the soils (Table 2). The sum of the exchangeable cations in the soils was GBC Acrisol > Dzorwulu Vertisol > Adansam Lixisol > Lukweta Acrisol > Dompem Acrisol > Chitotokoloki Arenosol. Further, the urban garden soils (GBC Acrisol and Dzorwulu Vertisol) had > 50% base saturation compared to the other soils (Table 2).

**Table 1:** Properties of the original soils used (N = > 3 replicates).

Original soil	Bulk density	Sand	Silt	Clay	Texture	Soil pH	Total C	Av. P
	(g cm^− 3^)	(%)				(Water)	(g kg^− 1^)	(mg kg^− 1^)
Adansam Lixisol	1.4	91	2	7	Sand	6.4	9.9	19.2
Chitotokoloki Arenosol	1.5	92	2	6	Sand	6.5	0.5	16.5
Dompem Acrisol	1.2	74	16	11	Loamy sand	4.6	16.9	4.2
Dzorwulu Vertisol	2.2	42	12	46	Clay	7.6	8.0	162.7
GBC Acrisol	1.6	74	7	19	Sandy loam	7.2	12.9	200.3
Lukweta Acrisol	1.3	81	10	9	Loamy sand	6.7	1.9	19.4

**Table 2:** Delta pH, CEC, exchangeable cations and base saturation of the original soils (N = > 3 replicates).

Soil	Delta pH	CEC (cmol_c_ kg^− 1^)	Exchangeable cations (cmol_c_ kg^− 1^)				Base saturation (%)
			Ca	Mg	Na	K	
Adansam Lixisol	0.1	7.6#	4.6	1.2	0.3	1.2	-
Chitotokoloki Arenosol	2.6	4.5	0.9	0.2	0.1	0.1	27.1
Dompem Acrisol	-0.6	3.9#	1.9	1.0	0.2	0.0	-
Dzorwulu Vertisol	-0.2	16.4	6.6	1.5	0.4	0.4	53.9
GBC Acrisol	-0.4	12.8	9.1	1.3	0.7	0.5	90.3
Lukweta Acrisol	1.3	9.5	3.1	0.8	0.6	0.1	47.9

# ECEC with no base saturation data.

#### Organic soil amendments

Each of the materials used for experimental TP formation exhibited unique properties. The biochar had neutral pH values, while urine and ash were alkaline (Table 3). The CEC of the charcoal was 55.8 cmol_c_ kg^− 1^, whereas that of the biochar was 20.3 cmol_c_ kg^− 1^. All the materials provided over 100 g kg^− 1^ total C except for the urine. The urine was the highest contributor of total P, followed by poultry manure and biochar, while the rest of the materials had < 1 g kg^− 1^ P. The manures, kitchen leftovers, biochar, ash and urine contributed over 50 mg kg^− 1^ Ca, while pig manure, main test kitchen leftovers, and urine provided over 50 mg kg^− 1^ Mg and Na compared to the others. The materials provided over 50 mg kg^− 1^ K except for charcoal (Table 3).

**Table 3:** Properties of the manures and residues used (N = > 3 replicates).

Material	pH	Total C	Total P	Ca	Mg	Na	K
	(Water)	(g kg^− 1^)		(mg kg^− 1^)			
Biochar^1^	6.6	302	1.6	240	96	269	195
Charcoal	6.8	302	0.3	30	4	6	26
Kitchen leftover	NM	368	0.3	165	35	30	293
Pig manure	NM	334	0.6	1285	219	1310	755
Poultry manure	NM	278	2.5	32	5	6	55
Urine*	9.0	2	34.6	67	64	105	159
Wood ash^2^	12.7	104	0.2	85	15	8	70

Data source: ^1^MacCarthy et al. (2020); ^2^Neina et al. (2020); *Units in mg or g/L; NM: not measured.

### Properties of the Terra Preta

#### Soil pH and delta pH

**Fig. 1:**
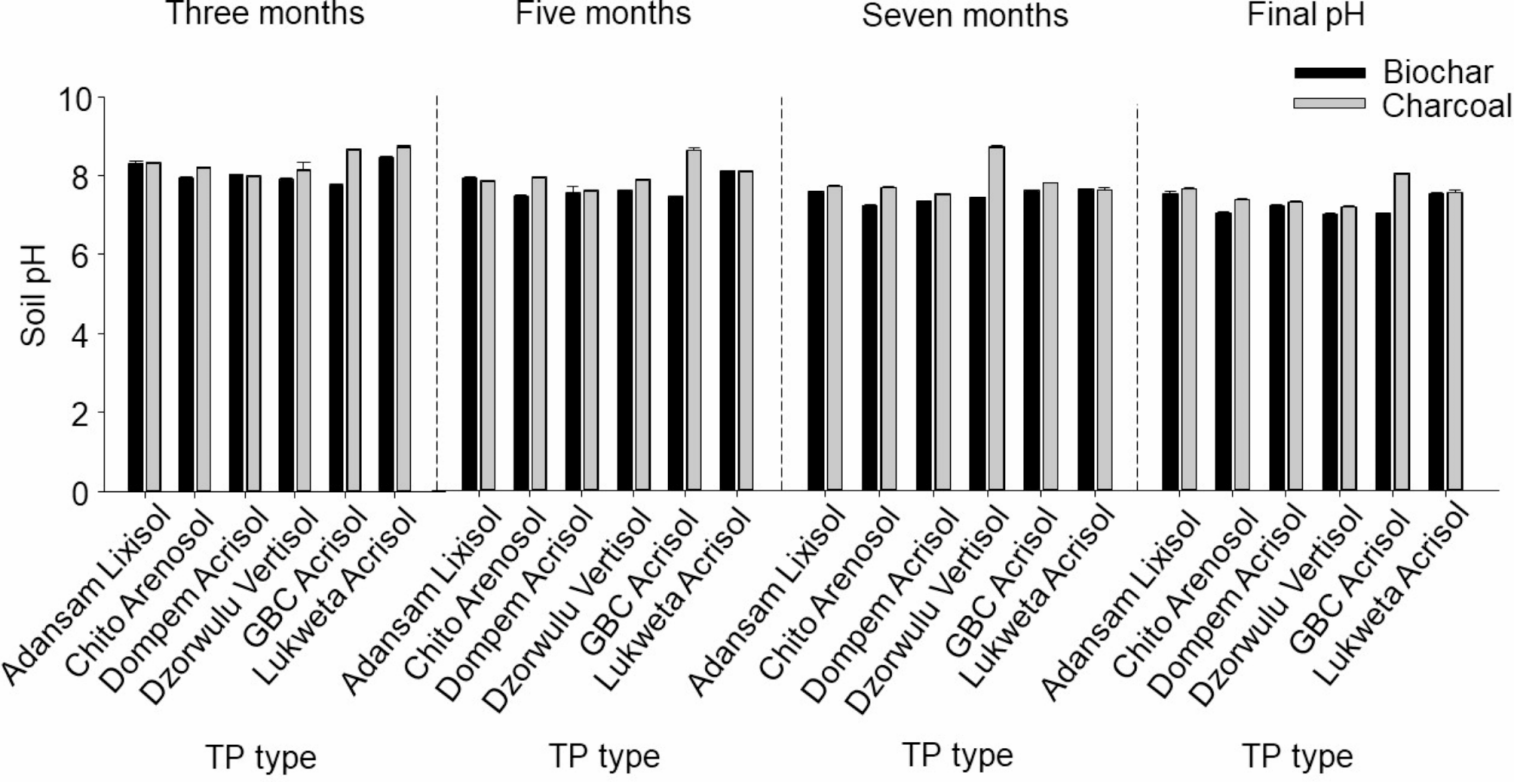
Temporal development of pH of the Terra Preta mixture from three, five, and seven months of incubation, and the final pH (*N* = 3 ± Standard error of means).

After three months of amendment and incubation, the pH of the TP mixture increased to values between 7.7 and 8.5 for biochar and 8.0 and 8.7 for charcoal (Fig. 1). During the experimental period, the pH decreased marginally from three to five months, beginning with a decrease by 0.3 to 0.4 pH units for biochar and up to 0.7 pH units for charcoal TP (Fig. 1). From this time to the seventh month, it decreased by 0.1 to 0.5 pH units for biochar and 0.1 to 0.8 pH units for charcoal, except for those of the urban garden soils. Charcoal consistently produced the greatest effect on pH from the third to the seventh month but dominated in the final month (Fig. 1). At the end of the experiment, there was a mix of decreases and increases in both the biochar and charcoal TPs. The final pH showed that the more neutral or alkaline pH of the urban garden soils, Dzorwulu Vertisol and GBC Acrisol, decreased by 0.4 and 0.03 pH units, respectively, from the original pH, whereas the original pH of the acidic soils increased by 0.7 to 2.8 pH units in the biochar TP. Conversely, the original pH of the Dzorwulu Vertisol decreased by 0.2 pH units, while other soils’ pH increased by 1 to 2.9 pH units in the charcoal TP (Table 1; Fig. 1). In each case, the greatest effect was observed in the Dompem Acrisol, particularly for charcoal. Unlike the pH, the ∆pH presented some remarkable trends: (a) the original negative ∆pH values of Dompem Acrisol and Dzorwulu Vertisol became positive after incubation for both biochar and charcoal, (b) the original positive ∆pH values of Adansam Lixisol became negative (-0.17, -0.13) after incubation, (c) the original negative ∆pH values of GBC Acrisol remained negative but reduced by 0.31 for biochar and 0.11 for charcoal, (d) the relatively high positive original ∆pH values of Chitotokoloki Arenosol and Lukweta Acrisol reduced drastically by almost a similar margin to almost zero for both biochar and charcoal (Tables 2 and 4).

**Table 4:** Delta pH, CEC, exchangeable cations, and base saturation of the biochar and charcoal Terra Preta.

Terra Preta	Delta pH	CEC (cmol_c_ kg^− 1^)	Exchangeable cations (cmol_c_ kg^− 1^)				% Base saturation
			Ca	Mg	Na	K	
*Biochar*							
Adansam Lixisol	-0.2	30.0 a	6.4 a	5.18 a	2.8 a	13.8 a	94.0 a
Chitokoloki Arenosol	0.2	28.7 b	5.7 b	4.92 b	2.6 b	13.6 a	93.7 b
Dompem Acrisol	0.2	28.8 b	6.3 a	5.09 c	2.5 c	13.1 a	93.8 b
Dzorwulu Vertisol	0.1	41.8 c	8.2 c	3.79 d	5.1 d	22.8 b	95.7 c
GBC Acrisol	-0.1	37.8 d	7.7 d	3.42 d	4.5 e	20.4 b	95.3 d
Lukweta Acrisol	0.0	25.8 e	5.6 b	4.94 b	2.2 f	11.3 c	93.0 e
*CV*_*pooled*_ *(%)*	*61.3*	*18.2*	*15.2*	*15.5*	*34.8*	*27.5*	*1.0*
*p-values*	*-*	*0.006*	*0.007*	*0.006*	*0.006*	*0.008*	*0.006*
*Charcoal*							
Adansam Lixisol	-0.13	29.4 a	6.0 a	5.9 a	2.6 a	13.2 a	93.9 a
Chitokoloki Arenosol	0.2	36.9 b	14.7 b	7.8 b	2.2 b	10.4 b	95.1 b
Dompem Acrisol	0.1	30.7 a	6.8 a	5.6 a	2.5 a	14.0 a	94.2 a
Dzorwulu Vertisol	0.2	42.5 c	10.2 c	4.2 c	4.8 c	21.5 c	95.8 c
GBC Acrisol	-0.3	37.5 d	6.9 d	2.9 d	4.4 c	21.5 c	95.2 d
Lukweta Acrisol	0.0	31.5 e	6.7 d	5.4 a	2.7 a	15.0 a	94.3 e
*CV*_*pooled*_ *(%)*	*57.2*	*13.7*	*37.0*	*29.2*	*33.1*	*26.9*	*0.70*
*p-values*	*-*	*0.006*	*0.009*	*< 0.001*	*0.009*	*0.006*	*0.007*

Data with means (*N* = 3) in columns and pooled coefficient of variation (CV_pooled_) under each group followed by different letters depict significant differences at 5% significance level.

#### Cation exchange capacity, basic cations, and base saturation

The CEC varied from 26 to 42 cmol_c_ kg^− 1^ (*p* = *0.006*) and increased by 2.5 to 7.4-fold of the original values for the biochar TP (Tables 2 and 4). In the charcoal TP, the CEC varied from 30 to 43 cmol_c_ kg^− 1^ (*p* = *0.006*) and increased by 2.6 to 8.2-fold. The highest values were found in the urban garden soils. For absolute effects, the charcoal TP produced the highest CEC values except for Adansam Lixisol and GBC Acrisol. For the extent of impact, charcoal had the highest magnitude except for the Adansam Lixisol (Tables 2 and 4). For the exchangeable cations, all the soils gained 1.6 to 4.82 cmol_c_ kg^− 1^ more Ca for biochar (*p* = *0.007*) and 1.4 to 13.8 cmol_c_ kg^− 1^ more Ca for charcoal Terra Preta (*p* = *0.009*), except for the GBC Acrisol which lost 1.4 and 2.2 cmol_c_ kg^− 1^ Ca. In both biochar and charcoal, the Chitotokoloki Arenosol had the highest magnitude of increase in the exchangeable Ca content. Exchangeable Mg contents varied from 3.4 to 5.2 cmol_c_ kg^− 1^ for biochar (*p* = *0.006*) with enhancements of 25-fold in the Chitotokoloki Arenosol but 2.5 to 6.2-fold for the biochar TP. For the charcoal TP, Mg contents varied from 2.9 to 7.8 cmol_c_ kg^− 1^ (*p* < *0.001*) with enhancements of 39-fold in the Chitotokoloki Arenosol but 2.3 to 6.8-fold for the charcoal TP, suggesting a greater effect of the charcoal residues on the Mg contents (Tables 2 and 4). Exchangeable Na also varied from 2.2 to 5.1 cmol_c_ kg^− 1^ (*p* = *0.006*), increasing by 3.6 to 26-fold, with the largest in the Chitotokoloki Arenosol for biochar TP. In the charcoal TP, it varied from 2.2 to 4.8 cmol_c_ kg^− 1^ (*p* = *0.009*), increasing by 4.4 to 21.7-fold with the largest in the Chitotokoloki Arenosol for charcoal TP (Tables 2 and 4). Exchangeable K had the largest cation gained from the amendments. It varied from 11.3 to 22.8 cmol_c_ kg^− 1^ (*p* = *0.008*), increasing by 11.5 to 136-fold largest in the Chitotokoloki Arenosol biochar TP. Conversely, the contents varied from 10.3 to 21.5 cmol_c_ kg^− 1^ (*p* = *0.006*) and increased by 10 to 21-fold with the largest in the urbans garden soils for charcoal TP. Overall, the exchangeable cation contents were in the order K > Ca > Mg > Na.

The base saturation was not calculated for the Adansam Lixisol and Dompem Acrisol because of the pH values, resulting in missing values. For other soils, the base saturation ranged from 93 to 96% and 94–96% for the biochar and charcoal TP, respectively. The increase in base saturation was in the order Chitotokoloki Arenosol > Lukweta Acrisol > Dzorwulu Vertisol > GBC Acrisol (Tables 2 and 4). A few of the properties correlated with each other, including positive Spearman correlations between CEC and base saturation (*r* = 0.99, *p* < 0.001) and negative Spearman correlations between pH and base saturation (*r* = -0.54, *p <* 0.021), and pH and CEC (*r* = -0.53, *p <* 0.023) for biochar. A negative Spearman correlation was found between pH and delta pH (*r* = − 0.93, *p* < 0.001), positive correlations between the sum of basic cations and CEC (*r* = 1.0, *p* < 0.00001) and CEC or sum of basic cations and base saturation (*r* = 0.99, *p* < 0.001) for charcoal.

**Fig. 2:**
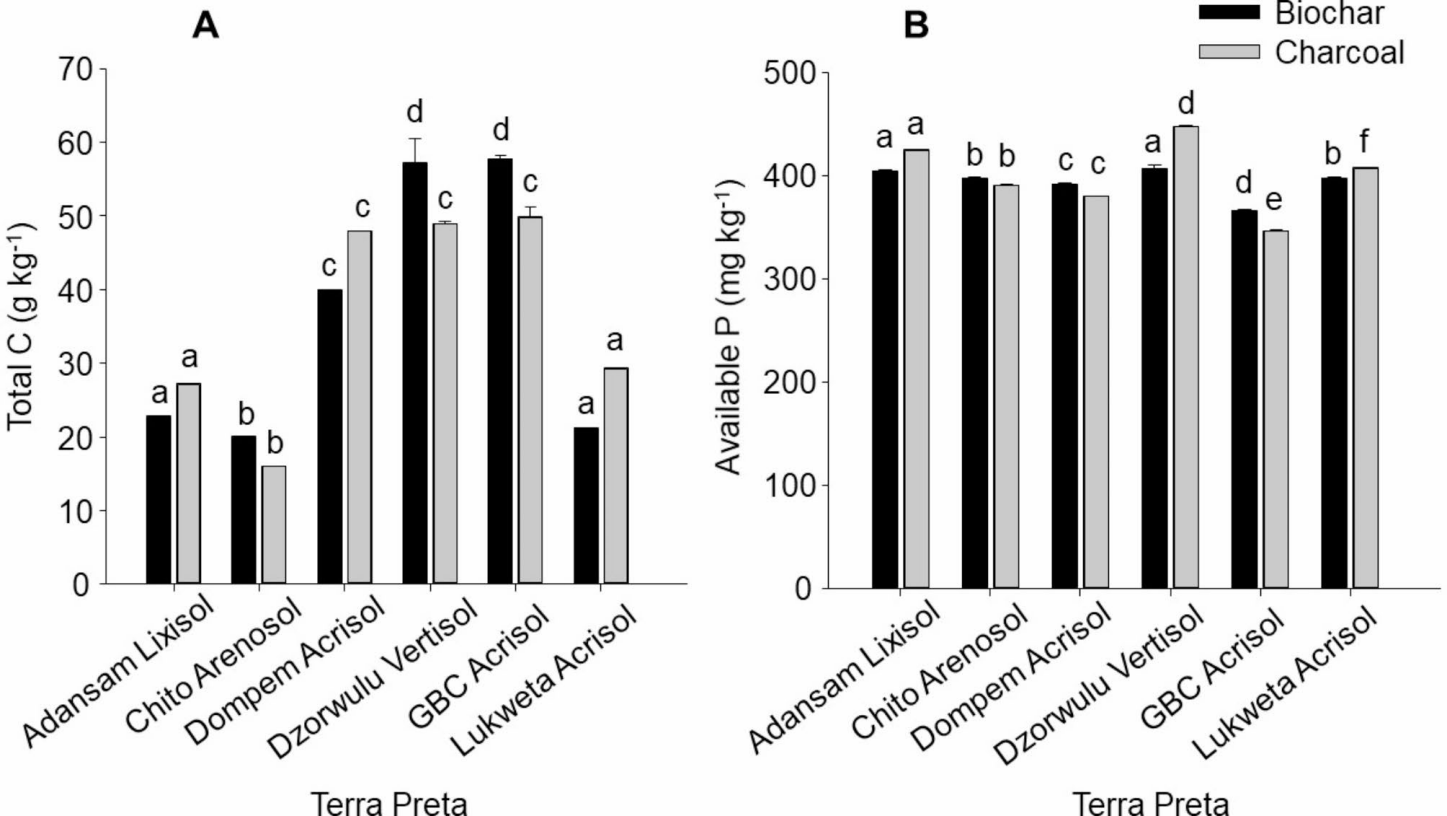
Total C (**A**) and plant-available P (**B**) contents of the biochar (*p = 0.008*) and charcoal (*p = 0.005*) Terra Preta (*N* = 3 ± Standard error of means). Bars of the same colour followed by different letters depict significant differences at 5% significance level.

Total C contents varied from 20 to 58 g kg^− 1^ for biochar (*p = 0.008*) and from 16 to 50 g kg^− 1^ for charcoal (*p = 0.005*) TP, with the highest and lowest contents in the GBC Acrisol and Chitokoloki Arenosol (Fig. 2A), respectively. Generally, the Zambian soils (Chitokoloki Arenosol and Lukweta Acrisol) gained more total C. For instance, Chitokoloki Arenosol benefited the most, reaching 40-fold for the biochar and 32-fold for charcoal TP, followed by Lukweta Acrisol with more total C from charcoal than biochar. These were followed by Dzorwulu Vertisol and GBC Acrisol with 6 to 7-fold more total C from biochar, and Adansam Lixisol and Dompem Acrisol with 2.3 to 2.8-fold more total C from charcoal (Fig. 2A). The plant-available P contents ranged from 366 to 407 mg kg^− 1^ for biochar (*p* = *0.008*) and from 347 to 448 mg kg^− 1^ for charcoal TP (*p* = *0.005*). The plant-available P of the Dompem Acrisol increased by 93-fold for biochar and 90-fold for charcoal TP (Fig. 2B). The rest of the gains followed the order Chitokoloki Arenosol > Adansam Lixisol > Lukweta Acrisol > Dzorwulu Vertisol > GBC Acrisol with small margins between biochar and charcoal TP.

## Discussion

The TP concept and the philosophy of pre-Colombian Amazonia enhanced the fertility of existing poor soils, transforming them into more productive ones, as confirmed by previous research on the Amazonian TP^4,37–39^. This study of experimental TP formation under coastal savannah climatic conditions and on soils from different agroecological zones and the results obtained proved that the replication of TP is achievable, particularly in the tropics, once there is commitment and perseverance from all relevant stakeholders. Like the Amazonian TP, biochar and charcoal residues, along with decomposable wastes, produced darker and more fertile soils, having strong aggregation of the soil particles, suggesting signs of soil structural development. These observations can be attributed to (i) the quality of biochar, food leftovers and human and animal excrements, and the soils used (Tables 1 and 2), and (ii) their microbial interactions with the soils during the “pedo-decomposition” process. These interactions lead to partial organic matter decomposition, releasing plant-available nutrients as immediate fertilizers while stabilizing the remaining organic matter via organo-mineral associations or due to chemical recalcitrance^4^. Specific pedogenic processes behind these interactions include: (a) *Cumulization* involving the additions of materials; (b) *Decomposition* and *turbation* of material by human beings as seen in this study and soil organisms^6,40^; (c) *Humification* to form humins and humic acids (HA) and fulvic acids (FA) associated with increased HA: FA ratios^28,41^; (d) *Organo-mineral complexation* where bivalent cations from ash and biochar interact through cation bridging with functional groups of the humic acids at elevated pH values enhancing the deprotonation of carboxylic groups bound to soil minerals^6,12,42^; (e) *Melanization* where soils are darkened by biological homogenization of biochar, SOM and mineral fractions^40,43^; (f) Slow biotic and abiotic oxidation of biochar edges which produces reactive carboxylic groups, increases nutrient-holding capacity and the potential of organo-mineral complex formation^3,12,44^.

Most studies suggest that the Amazonian TP contains significantly higher soil pH, Ca, Mg, P, K, base saturation, organic matter, and biological activity than adjacent soils^4^. Falcao et al.^13^, German^7^, and McCann^45^ suggested that total C, total P, and Ca are the three indicators of the Amazonian TP. The relevant elemental signatures of the Amazonian TP observed by Barbosa et al.^46^ were total P, Ca, K, and Mg, whereas those of da Costa et al.^11^ were total P, Mg, and Ca. This study’s TP is characterized by relatively high total C, pH, K, Ca, Mg, Na, base saturation, and available P. It is observed that each material used contributed uniquely to all the properties of the TP enhancing the properties by several folds compared to the original soil properties. These observations contrast the enrichment factors of 1.6 and 15.9-fold observed by Barbosa et al.^46^ for most elements. They, however, fall within values reported by Cunha et al.^41^ and Liang et al.^32^, and were attributed to the different wastes used since they provided over 100 g kg^− 1^ total C except for the urine.

Much of the total C in the TP can be attributed to the chars (Table 3; Fig. 2), which contain more stable C forms than the decomposable wastes and manures. None of the chars had absolute overriding effects on all the soils (Fig. 2), although the contents were much lower than those found in other studies^33,35^. Instead, there appeared to be a non-statistical interactive effect where the fine-textured soils appeared to protect some of the chars in some cases. Consequently, the final C contents in each soil type after incubation may be attributed to specific soil conditions that affect the decomposition processes. Plant-available P also varied with soil type with no marked effect of either of the chars. The plant-available P contents were similar to or lower than those of Lima et al.^28^, but were higher than those of da Costa et al.^11^, Falcão et al.^13^, and Macedo et al.^40^. Although biochar is known to be a major source of soil P^47–49^, the P was mainly supplied by urine, followed by poultry manure and biochar. Moreover, the P-supplying capacity of human excrement contrary to the P supply of biochar was recognized^10^.

The relatively high soil pH is characteristic of the Amazonian TP as found 0.02 to 2.9 pH units increases for both biochar and charcoal TP. In this study, however, the charcoal had a greater effect on the pH than the biochar. These values are higher than those often encountered in most TP^10,13,28^ and reflect the effect of the alkalinity of biochar, charcoal, and the waste ash. This alkalinity, and its effect have been reported by previous studies^26,50,51^. Tryon^25^ and Glaser et al.^12^ also reported earlier that hardwood biochar are more effective in reducing soil acidity. Moreover, it is also possible that the ash content used in this study was much higher than those present in Amazonian TP. Although hydrolyzed urine has high pH, it does not persist in soil after application because nitrification of the ammonium releases acidity to offset any pH increase^52^. The pH had a strong negative correlation with ∆pH (*r = -0.93*, *p < 0.001*) but only in the charcoal TP. Therefore, the ∆pH values encountered were either negative, zero or positive and differed slightly from those reported by de Aquino et al.^53^ and Falcao et al.^13^, who found only negative and zero charges in surface and subsurface Amazonian TP, respectively. These marked differences may be associated with complex interactions between the buffering capacity, point of zero charge, mineralogy, and the quality of organic matter of each soil type^54,55^.

Unlike the strong negative correlation between pH and ∆pH for the charcoal TP, the pH had a negative correlation with CEC (*r = -0.53*,*p < 0.023*) only for the biochar TP. These suggest that the chars had unique effects on the charge properties of the TPs. These dynamics may be responsible for the CEC of the experimental TP. The CEC values of 3 to 8-fold compared to the original soils were several folds higher than those of Macedo et al.^40^ and de Aquino et al.^53^. The magnitude of CEC enhancement of the original soils was much higher than those reported by Liang et al.^32^, who found about 2-fold CEC in the TP. These values were generally attributed to the char types. Specifically, charcoal had a stronger effect on the CEC of most of the soils than biochar (Table 4). Irrespective of the feedstock type, biochar produced at low temperatures possesses higher CEC^34,35^. Generally, at the same pyrolysis temperature, hardwood biochar has a higher CEC than non-hardwood biochar and imparts it to soils more effectively^12,25,35^. The rice husk biochar used in this study was pyrolyzed at 360 °C^56^, whereas the pyrolysis temperature of charcoal is between 360 and 470 °C^57^. These may explain the reasons for the difference in their CEC values.

Remarkably, the CEC further influenced the sum of basic cation contents and base saturation as seen in the strong positive correlations, but only for the charcoal TP. Overall, the magnitude of basic cation contents was K > Ca > Mg > Na. The charcoal had the greatest effect on Ca and Mg, whereas the effect of the biochar was only marginally observed in the exchangeable K contents. These values are similar to those found by Falcao et al.^13^ and Lima et al.^28^ but lower than those of Macedo et al.^40^, de Aquino et al.^53^, and Cunha et al.^41^. Consequently, the base saturation was high in the TP, ranging from 93 to 96%. Again, these values exceeded those of Macedo et al.^40^, de Aquino et al.^53^, Cunha et al.^41^, and Lima et al.^28^, but similar to those of Falcao et al.^13^. These differences could be caused by the effect of rainfall on the TP, which can lead to leaching losses of basic cations once conditions are favorable, considering the ecological location of the TP. In this study, the TP were protected from the impact of rainfall and thus retained the cations in the soils.

With the remarkable differences in the TP properties, the unique effects of each soil type cannot be discounted. First, a decrease and an increase in the original alkaline and acidic soils, respectively, are observed, followed by an improvement in the ∆pH of the more sandy soils. Then, a mixed pattern of effects can also be observed for Ca and Mg in the Chitotokoloki Arenosol and loamy Dompem Acrisol. These effects can be attributed to dilution effects, pedo-decomposition, and the buffering capacity of the native soil because buffering capacity, for instance, is controlled by soil organic C, clay mineralogy, clay content, cation valence, and ionic strength^58,59^. Previously, Tryon^25^ observed that pH increased more strongly in loamy and sandy than clay soils after charcoal addition. These contrasts the findings of this study as seen in the pH differences of the original and TP. It was observed that at the same application rate, 1.5-fold increase in the CEC of loamy sand than clayey soil^60^. Blanco-Canqui^61^ also observed that sandy soils benefit more from biochar applications than clayey soils. Notably, these variations are probably one of the reasons for the wide variabilities observed in TPs^62^, giving an opportunity for preferences, where many Amazonian farmers prefer clayey TP because of their high quality compared to sandy TP^7,14^.

As self-regenerating soils, the TP provide hope for soil resilience and sustainability for fragile soil systems. Their impressive fertility and productivity are proven to be associated with increased soil pH caused by the alkalinity of biochar, which makes essential nutrients such as Fe, Zn, Cu, Mn, Mo, and B more available for plant uptake. This is because of the key role of soil pH in plant nutrition^63^ and yield outcomes, particularly in acid soil conditions. In a previous review, Jeffery et al.^23^ observed that, generally, tropical soils benefit more from biochar than temperate soils mainly due to their effectiveness in neutralizing soil acidity. The TP pH values (≥ 7.2) of the could lead to some micronutrient deficiencies^63,64^. These pH levels are associated with the observed levels of basic cations and base saturation^64^. However, in external environments, dilution by rainfall could reduce the pH. The self-regenerating potential of TP has been observed in high biomass-producing environments such as Amazonia. It is uncertain if this is applicable to low biomass-producing and semi-arid areas that can guarantee short fallows for the regeneration of the Amazonian TP and the off-site TP as practiced in Amazonia. This off-site test of TP formation assumes that on-site TP formation is also feasible, particularly when consciousness, commitment, and perseverance become an integral part of soil management choices made by researchers, policy makers, and farmers. Further research is required to understand the environmental, socio-cultural and sustainability implications of on-site TP formations.

Despite the high fertility of the TP, there have been questions regarding its vulnerability to degradation. Interviews with farmers who actively cultivated and made a livelihood from the TP confirmed that degradation occurs after a few years of cultivation depending on the crop grow due to differences in nutrient extraction, and levels of intensification^7,15^. For instance, it was observed that the fertility of the TP declined after six years of continuous cultivation of heavy feeders such as cassava^7^. Also, Junqueira et al.^15^ found that over-intensification of watermelon cultivation degraded the Amazonian TP. Despite this challenge, the farmers and relevant stakeholders uphold TP as a symbol of sustainability, given the wide range of crops that can be grown. They also observed a huge potential of TP to recuperate or self-regenerate if left undisturbed for about a year^7,15,45^. Consequently, they devised strategies such as reduced intensification by introducing one to two-year fallows, the incorporation of crop rotation, and occasional application of organic matter to curb degradation^7,15^. These could form part of future considerations in replicating and sustaining TP in suitable environments.

## Conclusions

This study successfully experimented off-site formation of twelve different TP under coastal savannah climatic conditions within nine months. The TP were characterized by relatively high total C, pH, K, Ca, Mg, Na, base saturation, and available P based on the Amazonian TP indicators. The properties reflected unique interactions among the chars, decomposable wastes, and the soils. Although the TP pH values fall slightly outside the productive range of 5.5 and 6.5, it is envisaged that this may not occur under field conditions due to the effects of rainfall and plant nutrient uptake. Nonetheless, this calls for further research under various climatic and soil conditions to ascertain appropriate specific material requirements for different soil types. The study has environmental, socio-cultural, and socio-economic implications. Environmentally, it promotes effective management, recycling, and upcycling of domestic waste streams and achieve some climate controls. However, socio-cultural barriers to the use of human excrements in certain cultures need to be broken. Nonetheless, TP could improve the socio-economic status of farmers by enhancing their livelihoods. The study suggests the potential for on-site TP formation. But the drawbacks are (1) the study was carried out in enclosed environmental conditions without exposure to external environmental forces and plant uptake, which could affect the final properties. (2) Huge quantities of materials would be required for large farm sizes, which can be a huge financial challenge for many small holder farmers. (3) Blanket rates of the chars and ash were used, which may not be suitable for all soil types as seen in the different TP. Overall, the TP formation requires consciousness, commitment and perseverance with focus on the goal of achieving high productivity.

## Materials and methods

### Material resources used

#### Soils

**Fig. 3:**
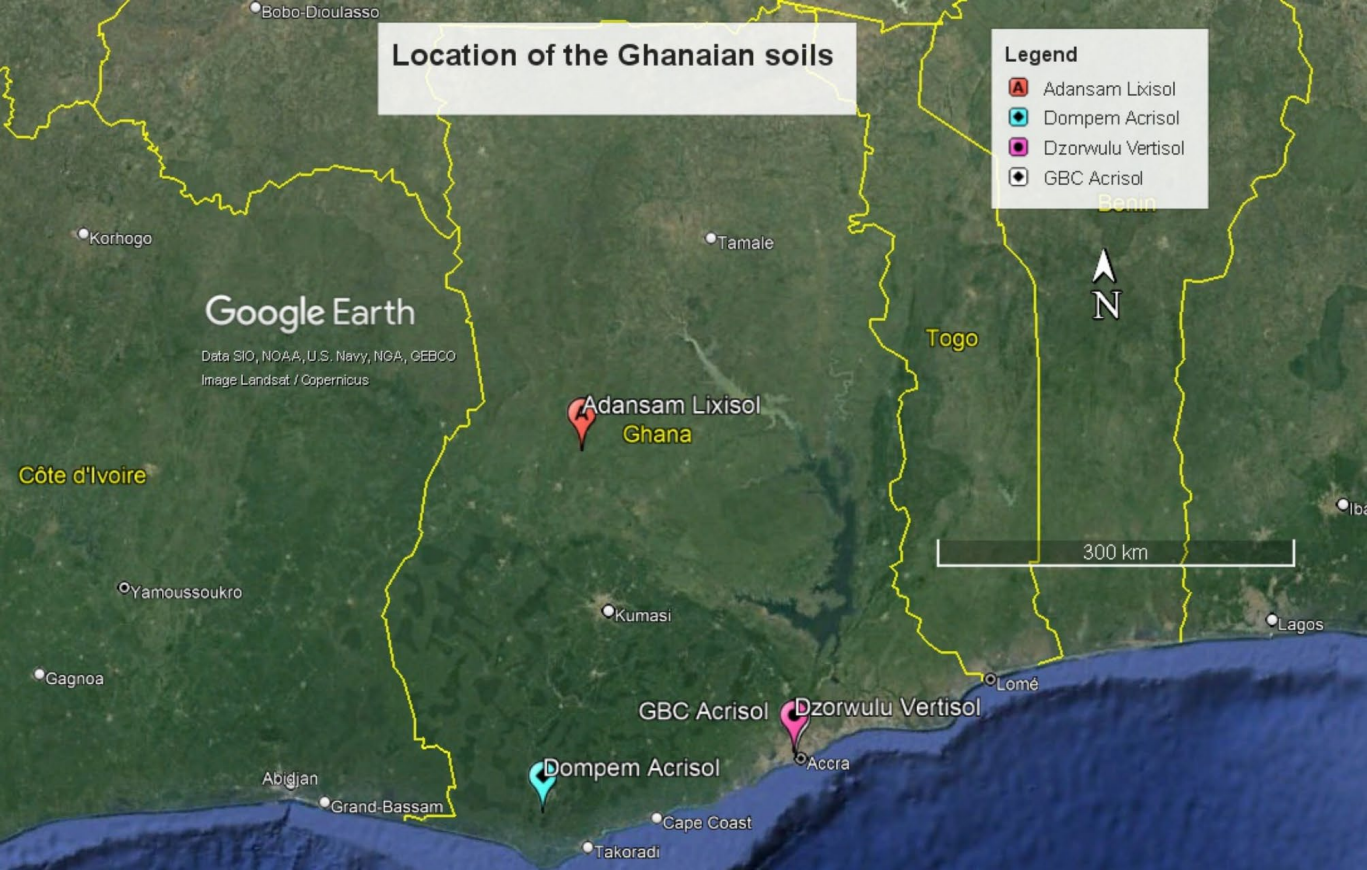
Major locations of the Dzorwulu Vertisol, GBC Acrisol, Adansam Lixisol, and Dompem Acrisol in Ghana.

**Fig. 4:**
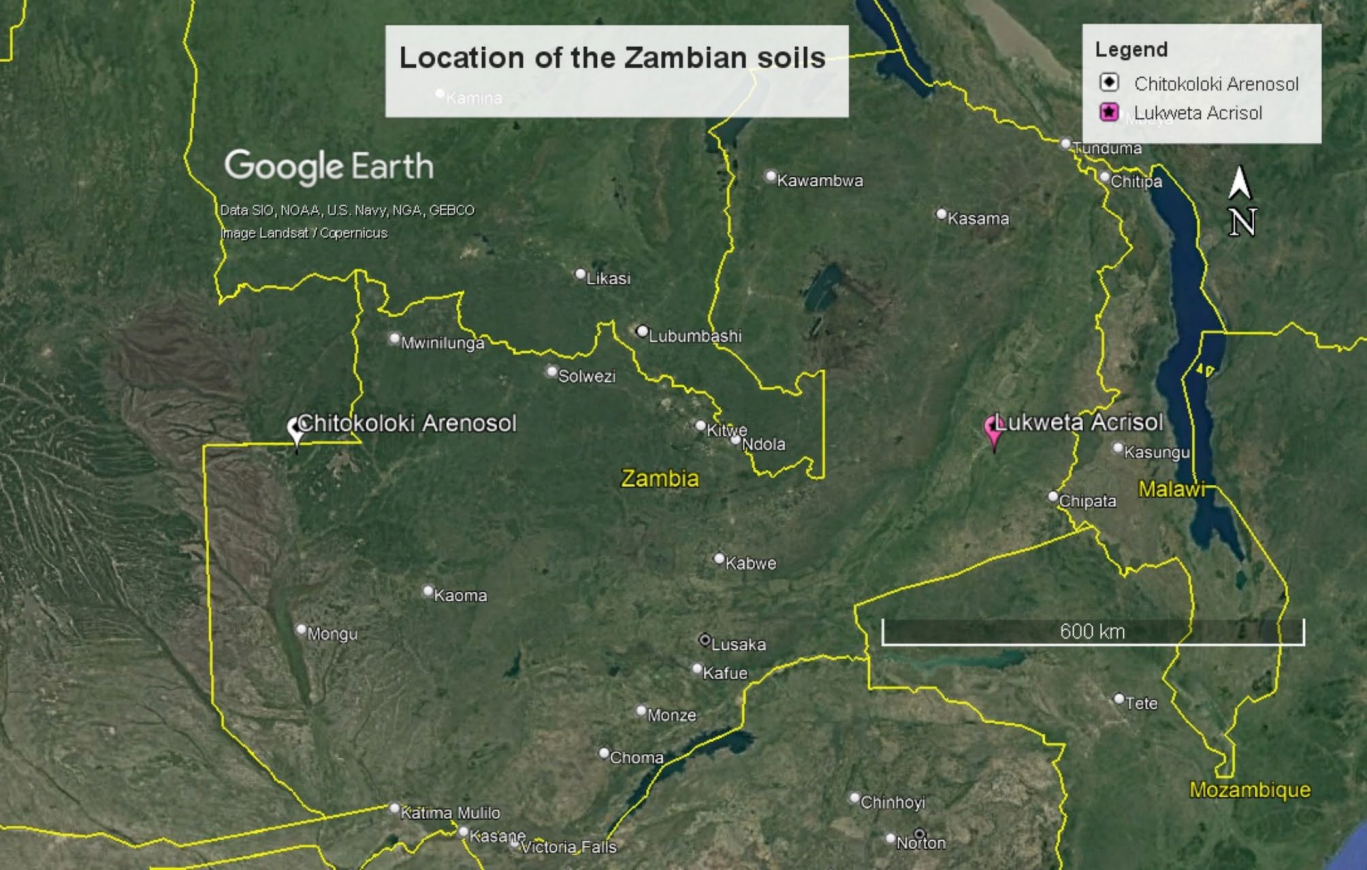
Major locations of the Chitokoloki Arenosol and Lukweta Acrisol in Zambia.

The experiment was conducted using four Ghanaian and two Zambian soils. The Ghanaian soils (Fig. 3) were a Vertisol sampled from the Dzorwulu Plant Pool and an Acrisol from the Ghana Broadcasting Corporation (GBC) areas, both from urban gardens in Accra. The soils had been cultivated under four seasons of rotational vegetable production for over 30 years. The other soils were obtained from five and ten-years cultivated farms comprising Lixisols from Adansam in the forest-savannah transition and Acrisols from Dompem in the forest zones of Ghana^65^. The soils are, herein, referred to as Dzorwulu Vertisol, GBC Acrisol, Adansam Lixisol, and Dompem Acrisol. The Zambian soils (Fig. 4) were Arenosols from Chitokoloki in the high rainfall agro-ecological zone and Acrisols from Lukweta in the medium rainfall zone of Zambia (Neina, unpublished). In this study, they are called Chitokoloki Arenosol and Lukweta Acrisol. All the soils except the urban garden soils were collected as part of the UK Research and Innovation Global Challenges Research Fund Sentinel Project.

#### Biochar and organic residues

Beside the soils, the other materials used for the study include charcoal residues, rice husk biochar, kitchen leftovers, human urine, wood ash, pig manure, and poultry manure. The charcoal residues were collected from charcoal vendors at Madina, a suburb of Accra. The rice husk biochar was a leftover from a previous study^56^, where it was charred using a Japanese retort kiln under sparing oxygen supply and pyrolyzed at an average temperature of 360 °C for 65 min. The wood ash was also a leftover collected from urban food vendors in Accra, Cape Coast, Ho, Koforidua, Sekondi-Takoradi through a previous study^51^ on which some analysis were carried out. The kitchen leftovers (which comprised yam, plantain, vegetable peels and mixtures of fruit wastes) were collected from food joints on the University of Ghana campus. The author’s urine was collected and stored for at least three months in a tightly closed jar before use. The pig and poultry manures were collected from the Livestock and Poultry Research Centre of the University of Ghana and from a piggery in the neighborhood.

### Experimentation procedure

**Fig. 5:**
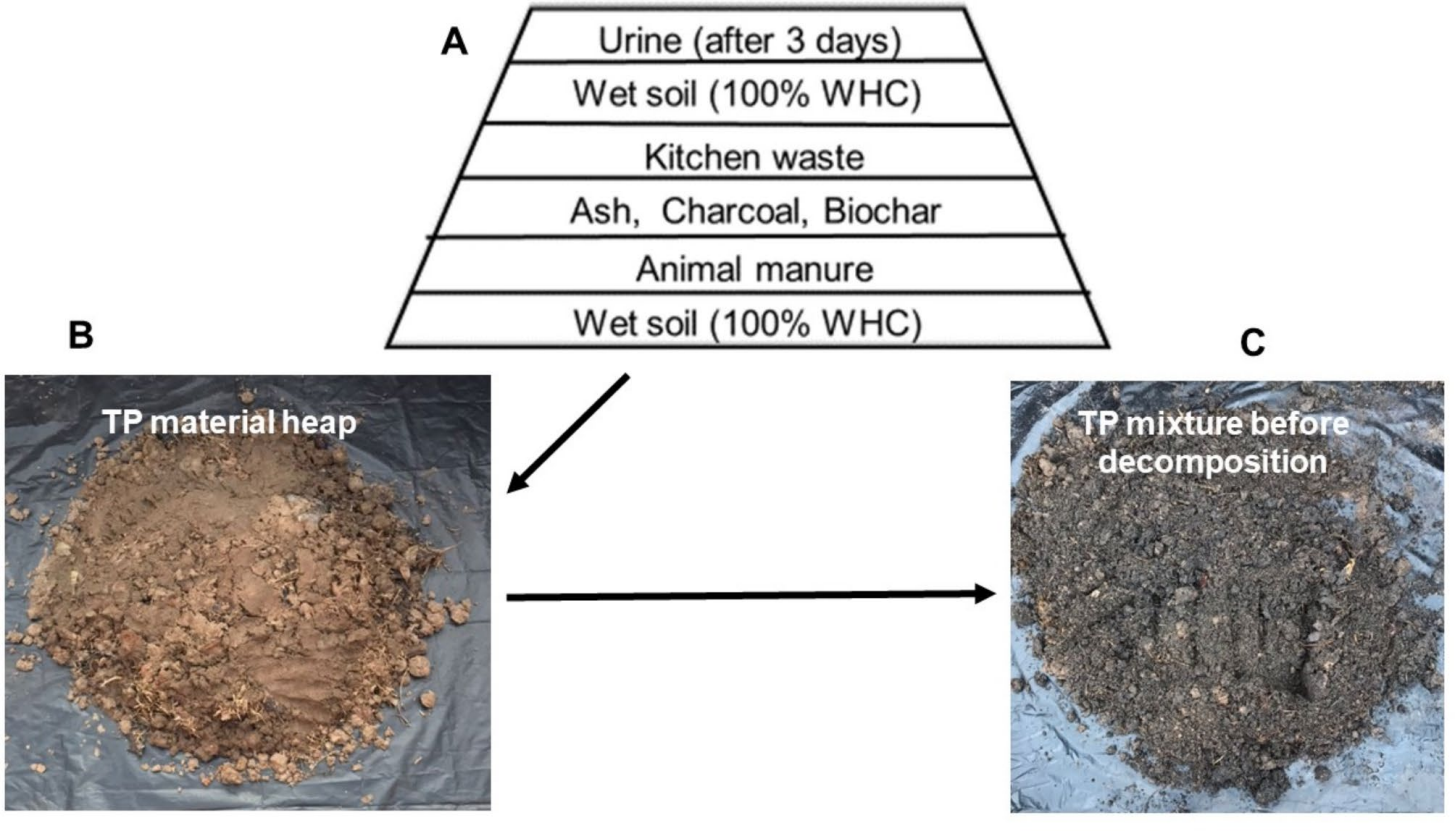
Sequence of material placement (**A**) during the four-day soaking and stabilization stage. The heap (**B**) was allowed to stabilize for three days after which urine was sprinkled, and left for another day before mixing (**C**).

The experimental TP formation was achieved by using the four Ghanaian and two Zambian soils, the rice husk biochar and charcoal residue to form biochar and charcoal TP. The weights of materials and soils used were based on the fractions indicated in Table 5. Specific quantities were a guide based on the findings of Glaser^10^. To start the incubation process, the soils were wetted to 100% water-holding capacity (WHC) by capillarity and allowed to equilibrate for 48 h. Subsequently, the kitchen leftovers were chopped into smaller chunks, where necessary, and packed in layers (Fig. 5). This order was used to enhance the soaking and stabilization of the material to kick-start the decomposition process. The layers were allowed to stabilize for three days after which urine was sprinkled and left for another day. Afterwards, the heap was mixed using a spade and packed into large plastic containers for decomposition to begin. Monitoring was done every other day to ensure adequate moisture required for the decomposition process. The mixture was occasionally turned to facilitate proper homogenization and decomposition. This process was repeated throughout for about three months. After three months, diluted urine was used to maintain the moisture content of the mixture for three consecutive periods till maturity. The pH of the mixture was measured from the third month and repeated monthly till termination in the tenth month of decomposition. The maturity of the produced biochar and charcoal TP were determined by visual examination for the disappearance of original organic matter fragments, stabilization of the pH during monitoring, and evidence of soil aggregate development.

**Table 5:** Composition of materials used for experimental Terra Preta formation.

Material	Quantity (w/w)	
	%	kg
Soil	50–60	10–12
Kitchen leftovers	15–30	3–5
Urine	5–7	1.4
Poultry/Pig manure	5	2.0
Ash	5	0.6
Rice husk biochar	5	1.0
Charcoal residues	5	1.0
Total weight (kg)	100	20

### Laboratory analyses

All soils used were collected to a depth of 20 cm, air-dried and passed through 2 mm before routine laboratory analysis were carried out. The soils were analyzed for routine properties as outlined in Neina and Agyarko-Mintah^65,66^, and Neina (unpublished). The properties measured include soil bulk density, particle size distribution and texture, pH (water and 1 M KCl), exchangeable cations, exchangeable acidity (where necessary) both at natural soil pH, total carbon (C), and plant-available P. The effective cation exchange capacity (ECEC) for soils with pH < 7 was calculated from the sum of exchangeable cations and exchangeable acidity contents.

The manures were air-dried, whereas the kitchen leftovers were oven-dried at 70 °C and milled. The milled fractions were sieved through 1 mm and 0.1 mm sieves for wet digestion (where necessary) and dry combustion, respectively. The elemental contents of the manure and biochar were measured using Atomic Absorption Spectrometer (AAS) (Perkin Elmer AAnalyst 800, Germany) and by UV/VIS spectrophotometry (Pharo 300 Spectroquant, Merck Millipore, Darmstadt, Germany) after di-acid digestion up to 200 °C. The total C contents of all the materials were measured by dry combustion of soil passed through a 0.5 mm sieve using a LECO Corporation TruMac Series CNS-2000 Analyzer (Leco Corporation, St. Joseph, MI, USA). The pH of the biochar and wood ash were measured in 1:10 biochar-water^56^ and 1:5 ash-water^51^ ratios, respectively.

At maturity, the TP were sampled, air-dried and sifted with a 2 mm sieve. The total C contents measured using dry combustion. Since, the pH of the TP had pH above 7, the effective CEC method was not applied. Instead, the CEC and exchangeable cations were measured using distillation and titration and AAS after extraction with 1 *M* NH4OAc buffered to pH 7, respectively. Plant-available P, instead of total P, was measured using Olsen P extraction method^67^ and measured at 710 nm on a UV/VIS spectrophotometer (Pharo 300 Spectroquant, Merck Millipore, Darmstadt, Germany) after coloration of the extracts with ammonium molybdate and ascorbic acid. This choice was made because of critical P levels in the existing soils.

### Statistical analysis

All the data generated were checked for normality using the Kolmogorov-Smirnov test to determine the appropriate statistical analysis. Unfortunately, the data were not normally distributed for two-way Analysis of Variance (two-way ANOVA). They were then split into those of biochar and charcoal and tested for normality using Shapiro-Wilk test before one-way ANOVA and Tukey HSD tests for mean comparison at 5% significant level for normally distributed data. At this level, most of the data, which were normally distributed and also had unequal variances even after transformation with logarithm and square-root, were tested using non-parametric Kruskal-Wallis with Mann-Whitney U tests at 5% significant level. Spearman’s correlation was conducted to assess relationships between properties. No statistical analysis was conducted on the soil pH and ∆pH data. The data were analyzed using SPSS version 20 (IBM^®^ SPSS^®^ Statistics, New York, USA). The graphs were produced with the help of Sigma Plot 13 (Systat Software Inc., San Jose, USA).

## Data Availability

The data will be made available upon request to the corresponding author.

## References

[1] Lal, R. et al. Managing soils for recovering from the COVID-19 pandemic. *Soil. Syst. ***4**, 46. 10.3390/soilsystems4030046 (2020).

[2] Bezerra, J. et al. The promises of the amazonian soil: shifts in discourses of Terra Preta and biochar. *J. Environ. Plann. Policy Manage. ***21**, 623–635. 10.1080/1523908X.2016.1269644 (2019).

[3] Glaser, B., Haumaier, L., Guggenberger, G. & Zech, W. The ‘Terra Preta’ phenomenon: a model for sustainable agriculture in the humid tropics. *Naturwissenschaften*. **88**, 37–41 (2001). 11302125 10.1007/s001140000193

[4] Glaser, B. & Birk, J. J. State of the scientific knowledge on properties and genesis of Anthropogenic Dark Earths in Central Amazonia (terra preta de Índio). *Geochim. Cosmochim. Acta*. **82**, 39–51 (2012).

[5] Lombardo, U. et al. Evidence confirms an anthropic origin of amazonian dark earths. *Nat. Comm. ***13**, 1–6. 10.1038/s41467-022-31064-2 (2022). 10.1038/s41467-022-31064-2 PMC9205880 35715390

[6] Glaser, B., Balashov, E., Haumaier, L., Guggenberger, G. & Zech, W. Black carbon in density fractions of anthropogenic soils of the Brazilian Amazon region. *Org. Geochem. ***31**, 669–678 (2000). -6380(00)00044 – 9.

[7] German, L. A. Unpublished PhD Thesis. University of Georgia (2001).

[8] Lehmann, J. et al. Nutrient availability and leaching in an archaeological Anthrosol and a Ferralsol of the Central Amazon basin: fertilizer, manure and charcoal amendments. *Plant. Soil. ***249**, 343–357. 10.1023/A:1022833116184 (2003).

[9] Neves, E. G., Petersen, J. B. & Bartone, R. N. & Da Augusto Silva, C. in *Amazonian Dark Earths* (eds. Lehmann, J., Kern, D.C., Glaser, B., Wodos, W.I.) 29–50 (Springer, Dordrecht, 2003).

[10] Glaser, B. Prehistorically modified soils of central Amazonia: a model for sustainable agriculture in the twenty-first century. *Philos. Trans. R Soc. Lond. Ser. B*. **362**, 187–196. 10.1098/rstb.2006.1978 (2007). 17255028 10.1098/rstb.2006.1978 PMC2311424

[11] Da Costa, M. L. & Kern, D. C. Geochemical signatures of tropical soils with archaeological black earth in the Amazon, Brazil. *J. Geochem. Explor. ***66**, 369–385 (1999). 10.1016/S0375-6742(99)00038-2

[12] Glaser, B., Lehmann, J. & Zech, W. Ameliorating physical and chemical properties of highly weathered soils in the tropics with charcoal - a review. *Biol. Fertil. Soils*. **35**, 219–230. 10.1007/s00374-002-0466-4 (2002).

[13] Falcão, N. P., Clement, C. R., Tsai, S. M., & Comerford, N. B. Pedology fertility, and biology of central Amazonian Dark Earths in Amazonian Dark Earths: Wim Sombroek’s vision (eds Woods, W. I., Teixeira, W. G., Lehmann, J., Steiner, C., WinklerPrins, A., & Rebellato, L.) 213–228 (Springer, Dordrecht, 2009).

[14] German, L. Ethnoscientific understandings of Amazonian Dark Earths in *Amazonian Dark Earths* (eds. Johannes, L., Kern, D.C., Glaser, B., Wodos, W. I.) 179–201 (Springer, Dordrecht, 2003).

[15] Junqueira, A. B., Almekinders, C. J. M., Stomph, T. J., Clement, C. R. & Struik, P. C. The role of amazonian anthropogenic soils in shifting cultivation: learning from farmers’ rationales. *Ecol. Soc. ***21**, 12. 10.5751/ES-08140-210112 (2016).

[16] Glaser, B. Terra Preta – Entstehung und Rolle für Klimaschutz und Stoffkreisläufe. In Warnsignal Klima: BodenLandnutzung (Lozán, J. L., 58, 380–387); 10.25592/warnsignal.kli- (2021).

[17] Jeffery, S., Verheijen, F., van der Velde, M. & Bastos, A. C. A quantitative review of the effects of biochar application to soils on crop productivity using meta-analysis. *Agric. Ecosyst. Environ. ***144**, 175–187. 10.1016/j.agee.2011.08.015 (2011).

[18] Gross, A., Bromm, T. & Glaser, B. Soil organic carbon sequestration after biochar application: a global meta-analysis. *Agron*. **11**, 2474. 10.3390/agronomy11122474 (2021).

[19] Edeh, I. G., Mašek, O. & Buss, W. A meta-analysis on biochar’s effects on soil water properties – new insights and future research challenges. *Sci. Total Environ. ***714**, 136857. 10.1016/j.scitotenv.2020.136857 (2020). 32018989 10.1016/j.scitotenv.2020.136857

[20] Gao, S., DeLuca, T. H. & Cleveland, C. C. Biochar additions alter phosphorus and nitrogen availability in agricultural ecosystems: a meta-analysis. *Sci. Total Environ. ***654**, 463–472. 10.1016/j.scitotenv.2018.11.124 (2019). 30447585 10.1016/j.scitotenv.2018.11.124

[21] Zhang, Q., Xiao, J., Xue, J. & Zhang, L. Quantifying the effects of biochar application on greenhouse gas emissions from agricultural soils: a global meta-analysis. *Sustainability*. **12**, 1–14. 10.3390/SU12083436 (2020).35136666

[22] Schmidt, H. P. et al. Biochar in agriculture – a systematic review of 26 global meta-analyses. *GCB Bioenergy*. **13**, 1708–1730. 10.1111/gcbb.12889 (2021).

[23] Jeffery, S. et al. Biochar boosts tropical but not temperate crop yields. *Environ. Res. Lett. ***12 **10.1088/1748-9326/aa67bd (2017).

[24] Major, J., Rondon, M., Molina, D., Riha, S. J. & Lehmann, J. Maize yield and nutrition during 4 years after biochar application to a Colombian savanna oxisol. *Plant. Soil. ***333**, 117–128. 10.1007/s11104-010-0327-0 (2010).

[25] Tryon, E. H. Effect of charcoal on certain physical, chemical, and biological properties of forest soils. *Ecol. Monogr. ***18**, 81–115. 10.2307/1948629 (1948).

[26] Yu, H. et al. Biochar amendment improves crop production in problem soils: a review. *J. Environ. Manage. ***232**, 8–21. 10.1016/j.jenvman.2018.10.117 (2019). 30466010 10.1016/j.jenvman.2018.10.117

[27] Steiner, C. et al. Long term effects of manure, charcoal and mineral fertilization on crop production and fertility on a highly weathered central amazonian upland soil. *Plant. Soil. ***291**, 275–290. 10.1007/s11104-007-9193-9 (2007).

[28] Lima, H. N., Schaefer, C. E., Mello, J. W., Gilkes, R. J. & Ker, J. C. Pedogenesis and pre-colombian land use of Terra Preta Anthrosols(Indian black earth) of Western Amazonia. *Geoderma*. **110**, 1–17. 10.1016/S0016-7061(02)00141-6 (2002).

[29] Neina, D. & Agyarko-Mintah, E. The Terra Preta Model soil for sustainable sedentary yam production in West Africa. *Heliyon*. **9**, e15896. 10.1016/j.heliyon.2023.e15896 (2023). 37168885 10.1016/j.heliyon.2023.e15896 PMC10165410

[30] Fairhead, J. & Leach, M. Amazonian Dark Earths in Africa? in *Amazonian Dark Earths: Wim Sombroek’s Vision* (eds. Woods, W.I., Teixeira, W.G., Lehmann, J., Steiner, C., WinklerPrins, A., Rebellato, L.) 265–278 (Springer Science + Business Media B.V, 2009).

[31] Asare, M. O. Anthropogenic dark earth: evolution, distribution, physical, and chemical properties. *Eur. J. Soil. Sci. ***73**, e13308. 10.1111/ejss.13308 (2022).

[32] Liang, B. et al. Black carbon increases cation exchange capacity in soils. *Soil. Sci. Soc. Am. J. ***70**, 1719–1730. 10.2136/sssaj2005.0383 (2006).

[33] Campos, P. et al. Chemical, physical and morphological properties of biochars produced from agricultural residues: implications for their use as soil amendment. *Waste Manage. ***105**, 256–267. 10.1016/j.wasman.2020.02.013 (2020). 10.1016/j.wasman.2020.02.013 32088572

[34] Rehrah, D. et al. Production and characterization of biochars from agricultural by-products for use in soil quality enhancement. *J. Anal. Appl. Pyrol. ***108**, 301–309. 10.1016/j.jaap.2014.03.008 (2014).

[35] Pariyar, P., Kumari, K., Jain, M. K. & Jadhao, P. S. Evaluation of change in biochar properties derived from different feedstock and pyrolysis temperature for environmental and agricultural application. *Sci. Total Environ. ***713**, 136433. 10.1016/j.scitotenv.2019.136433 (2020). 31954240 10.1016/j.scitotenv.2019.136433

[36] Schimmelpfennig, S. & Glaser, B. One step Forward toward characterization: some important material properties to distinguish Biochars. *J. Environ. Qual. ***41**, 1001–1013. 10.2134/jeq2011.0146 (2012). 22751042 10.2134/jeq2011.0146

[37] Briones, A. M. The secrets of El Dorado viewed through a microbial perspective. *Front. Microbiol. ***3**, 1–6. 10.3389/fmicb.2012.00239 (2012). 22866049 10.3389/fmicb.2012.00239 PMC3408238

[38] Kern, J., Giani, L., Teixeira, W., Lanza, G. & Glaser, B. What can we learn from ancient fertile anthropic soil (Amazonian dark earths, shell mounds, Plaggen soil) for soil carbon sequestration? *Catena*. **172**, 104–112. 10.1016/j.catena.2018.08.008 (2019).

[39] Palace, M. W. et al. Ancient amazonian populations left lasting impacts on forest structure. *Ecosphere*. **8**, e02035. 10.1002/ecs2.2035 (2017).

[40] Macedo, R. S., Teixeira, W. G., Corrêa, M. M., Martins, G. C. & Vidal-Torrado, P. Pedogenetic processes in anthrosols with pretic horizon (amazonian Dark Earth) in Central Amazon, Brazil. *Plos One*. **12**, e0178038. 10.1371/journal.pone.0178038 (2017). 28542442 10.1371/journal.pone.0178038 PMC5441626

[41] Cunha, T. J. F. et al. Soil organic matter and fertility of anthropogenic dark earths (Terra Preta De Índio) in the Brazilian Amazon basin. *Rev. Bras. Ciênc Solo*. **33**, 85–93. 10.1590/s0100-06832009000100009 (2009).

[42] Zhang, Q. et al. Water dispersible colloids and related nutrient availability in amazonian Terra Preta soils. *Geoderma*. **397**, 115103 (2021).

[43] Arroyo-Kalin, M. The amazonian formative: crop domestication and anthropogenic soils. *Diversity*. **2**, 473–504. 10.3390/d2040473 (2010).

[44] Novotny, E. H. et al. Lessons from the Terra Preta De Índios of the Amazon region for the utilisation of charcoal for soil amendment. *J. Braz Chem. Soc. ***20**, 1003–1010. 10.1590/S0103-50532009000600002 (2009).

[45] McCann, J. M. Before 1492: the making of the pre-columbian landscape: part I: the environment. *Ecol. Restor. North. Am. ***17**, 15–30 (1999).

[46] Barbosa, J. Z. et al. Elemental signatures of an Amazonian Dark Earth as result of its formation process. *Geoderma*. ** 361**, 114085; (2020). 10.1016/j.geoderma.2019.114085

[47] Sulemana, N., Nartey, E. K., Abekoe, M. K., Adjadeh, T. A. & Darko, D. A. Use of biochar-compost for phosphorus availability to maize in a concretionary ferric lixisol in northern Ghana. *Agronomy*. **11**, 359. 10.3390/agronomy11020359 (2021).

[48] Zhang, H. et al. Roles of biochar in improving phosphorus availability in soils: a phosphate adsorbent and a source of available phosphorus. *Geoderma*. **276**, 1–6. 10.1016/j.geoderma.2016.04.020 (2016).

[49] Deinert, L. et al. Poultry litter biochar soil amendment affects microbial community structures, promotes phosphorus cycling and growth of barley (*Hordeum vulgare*). *Eur. J. Soil. Biol. ***120**, 103591. 10.1016/j.ejsobi.2023.103591 (2024).

[50] Fidel, R. B., Laird, D. A., Thompson, M. L. & Lawrinenko, M. Characterization and quantification of biochar alkalinity. *Chemosphere*. **167**, 367–373. 10.1016/j.chemosphere.2016.09.151 (2017). 27743533 10.1016/j.chemosphere.2016.09.151

[51] Neina, D., Faust, S. & Joergensen, R. G. Characterization of charcoal and firewood ash for use in African peri-urban agriculture. *Chem. Biol. Technol. Agric. ***7 **10.1186/s40538-019-0171-2 (2020).

[52] Neina, D. & Dowuona, G. N. N. Short-term effects of human urine fertiliser and wood ash on soil pH and electrical conductivity. *J. Agric. Rural Dev. Trop. Subtrop*. **114**, 89–100 (2013).

[53] de Aquino, R. E. et al. Characteristics of color and iron oxides of clay fraction in Archeological Dark Earth in Apuí region, southern Amazonas. *Geoderma*. **262**, 35–44. 10.1016/j.geoderma.2015.07.010 (2016).

[54] Hemsi, P. S., Boscov, M. E. G. & Shackelford, C. D. Points of zero charge and adsorption for a Brazilian residual soil. In Proceedings of the Fourth International Congress of Environmental Geotechnics, Rio-De-Janeiro, Brazil, Aug (Vol. 1, pp. 1–15). (2002).

[55] Khawmee, K., Suddhiprakarn, A., Kheoruenromne, I. & Singh, B. Surface charge properties of kaolinite from Thai soils. *Geoderma*. **192**, 120–131. 10.1016/j.geoderma.2012.07.010 (2013).

[56] MacCarthy, D. S., Darko, E., Nartey, E. K., Adiku, S. G. K. & Tettey, A. Integrating biochar and inorganic fertilizer improves productivity and profitability of irrigated rice in Ghana, West Africa. *Agronomy*. **10**, 904. 10.3390/agronomy10060904 (2020).

[57] Coomes, O. T. & Miltner, B. C. Indigenous charcoal and biochar production: potential for soil improvement under shifting cultivation systems. *Land. Degrad. Dev. ***28**, 811–821. 10.1002/ldr.2500 (2017).

[58] Nelson, P. N. & Su, N. Soil pH buffering capacity: a descriptive function and its application to some acidic tropical soils. *Soil. Res. ***48**, 201. 10.1071/SR09150 (2010).

[59] Wang, X., Tang, C., Mahony, S., Baldock, J. A. & Butterly, C. R. Factors affecting the measurement of soil pH buffer capacity: approaches to optimize the methods. *Eur. J. Soil. Sci. ***66**, 53–64. 10.1111/ejss.12195 (2015).

[60] Ghorbani, M., Asadi, H. & Abrishamkesh, S. Effects of rice husk biochar on selected soil properties and nitrate leaching in loamy sand and clay soil. *Int. Soil. Water Conserv. Res. ***7**, 258–265. 10.1016/j.iswcr.2019.05.005 (2019).

[61] Blanco-Canqui, H. Biochar and soil physical properties. *Soil. Sci. Soc. Am. J. ***81**, 687–711. 10.2136/sssaj2017.01.0017 (2017).

[62] Madari, B., Cunha, T. J. F. & Soares, R. Organic matter of the anthropogenic dark earths of Amazonia. *Dyn. Soil. Dyn. Plant. ***5**, 21–28 (2011).

[63] Neina, D. The role of soil pH in plant nutrition and soil remediation. *Appl Environ Soil* 1–9; (2019). 10.1155/2019/5794869 (2019).

[64] Havlin, J. L. *Fertility reference module in earth systems and environmental sciences*. (Elsevier 2013). 10.1016/B978-0-12-409548-9.05162-9

[65] Neina, D. & Agyarko-Mintah, E. Duration of cultivation has varied impacts on soil charge properties in different agro-ecological zones of Ghana. *Land*. **11**, 1633. 10.3390/land11101633 (2022).

[66] Neina, D., Mwitwa, J. & Adolph, B. The nexus between soil Degradation and agricultural expansion in Zambia. Policy Brief Report (Sentinel / International Institute for Environment and Development, 2022).

[67] Olsen, S. R., Cole, C. V., Watanabe, F. S. & Dean, L. A. Estimation of available phosphorus in soils by extraction with sodium bicarbonate. USDA Circular 939. USDA, Government Printing Office, Washington DC. (1954).

